# Safety and efficacy profile of cyclin‐dependent kinases 4/6 inhibitor palbociclib in cancer therapy: A meta‐analysis of clinical trials

**DOI:** 10.1002/cam4.1970

**Published:** 2019-03-21

**Authors:** Linghong Guo, Yuanyuan Hu, Xi Chen, Qingfang Li, Benling Wei, Xuelei Ma

**Affiliations:** ^1^ State Key Laboratory of Biotherapy and Cancer Center West China Hospital, Sichuan University Chengdu China; ^2^ West China School of Medicine Sichuan University Chengdu China; ^3^ West China School of Stomatology Sichuan University Chengdu China; ^4^ General Hospital of Xuzhou Mining Group Xuzhou China

**Keywords:** CDK 4/6 inhibitor, efficacy, meta‐analysis, palbociclib, safety

## Abstract

**Background:**

Palbociclib is a small‐molecule, cyclin‐dependent kinase 4 and 6 inhibitor, which prevents phosphorylation of the retinoblastoma (Rb) protein and inhibits cell‐cycle progression from G1 to S phase. We performed this meta‐analysis to estimate the safety and efficacy of palbociclib in cancer patients from clinical trials.

**Methods:**

PubMed and EMBASE were searched for eligible studies. Adverse events (AE) of grade ≥3 and all‐grade (1‐5) were extracted to calculate event rates. Odds ratios (ORs) with 95% confidence interval (CI) were calculated to estimate the safety of palbociclib in endocrine treatment‐combined studies. A fixed effects model was used when homogeneity was low (*I* ^2^ ≤ 50%). A random effects model was adopted when there was a significant heterogeneity (*I* ^2^ > 50%). For efficacy endpoints, hazard ratio (HR) and 95% CI for progression‐free survival (PFS) or overall survival (OS) were extracted and analyzed.

**Results:**

Nine clinical trials representing 1534 patients were identified. The most frequently observed all‐grade adverse events (AEs) in patients treated with palbociclib were neutropenia (event rate: 68.1%), leukopenia (51.7%), fatigue (35.9%), anemia (34.7%), and thrombocytopenia (30.9%). The most common grade 3 or more toxicities were neutropenia (51.6%), leukopenia (29.4%), and thrombocytopenia (7.5%). Hematologic adverse events had high occurrence in the palbociclib group. The pooled analysis of survival outcomes suggested that palbociclib produced clinical benefits in breast cancers and Rb‐positive tumors. More specifically, palbociclib was associated with significant improvement of PFS (HR: 0.518, 95% CI: 0.444‐0.604) in the treatment of ER‐positive and HER2‐negative breast cancer.

**Conclusions:**

Hematologic adverse events were common in palbociclib‐treated cancer patients. Since palbociclib produced a higher PFS rate with a low serious complication rate, it can be a promising novel target therapy drug for treating ER‐positive and HER2‐negative breast cancer.

## 1 INTRODUCTION

Cyclin‐dependent kinases 4 and 6 (CDK 4/6) are activated by D‐type cyclins. By phosphorylating the retinoblastoma (Rb) protein, they can promote cell‐cycle progression from G1 to S phase.^1^, ^2^, ^3^, ^4^ Abnormalities during the progression from G1 to S phase are closely related to many malignancies.^5^, ^6^ CDK 4/6 were considered as a potential therapeutic target in tumors with functional Rb protein. Palbociclib is a bioavailable, highly specific inhibitor of CDK 4/6^3^ that prevents phosphorylation of the retinoblastoma (Rb) protein.^7^ Previous researches demonstrated that palbociclib had activity in reducing tumor growth in several Rb‐positive cell lines and xenograft models.^2^, ^8^ Furthermore, several clinical trials have suggested that palbociclib has antitumor activity in estrogen receptor (ER)‐positive breast cancer, genitourinary germ cell tumor, and some other retinoblastoma (Rb)‐positive tumors.^9^, ^10^, ^11^ From previous findings in clinical trials, we believed that CDK 4/6 inhibitor is potent in therapies for breast cancer and Rb‐positive tumor patients. At the same time, we should also pay more attention to the adverse effects caused by palbociclib or palbociclib‐based therapy. The adverse effects about palbociclib varied in different trials, which can be divided into treatment‐emergent adverse events (TEAE) and treatment‐related adverse events (TRAE). After reviewing these trials, we concluded that these therapies mainly manifested hematologic toxicity. To explore the potential clinical value of palbociclib, we conducted this study to find out the most meaningful adverse effects and efficacy outcomes of palbociclib and to direct further evaluation of the safety and efficacy of palbociclib.

## 2 METHODS

### 2.1 Search strategy

Clinical trials published in English considering this meta‐analysis were searched from PubMed database until 27 December 2017. The initial search keyword was “palbociclib” or “CDK 4/6 inhibitor” or “PD0332991.” The title and abstract of the identified studies were analyzed by two reviewers independently. Disagreements were resolved by consensus. In addition, references from selected publications were screened for potentially eligible studies. In addition, EMBASE was reviewed for following keywords including “palbociclib”, “CDK 4/6 inhibitor,” and “PD0332991” to avoid missing qualified studies (until 4 January 2018). Only the most complete and latest studies were included.

### 2.2 Study inclusion/exclusion criteria

Studies selected for final analysis should meet all of the following inclusion criteria: (a) prospective phase I, phase II, and III clinical trials used palbociclib in cancer patients; (b) data were available regarding the incidence of all‐grade adverse effects or grade ≥3 adverse effects or the survival outcome including overall survival (OS) or progression‐free survival (PFS); and (c) studies used palbociclib as a single‐agent or as combination therapy. The following exclusion criteria were applied: (a) repeated reports of same study group or repeated publications and (b) the study was not published in English.

### 2.3 Data extraction

Two independent investigators extracted the data needed from the selected studies, with disagreements resolved by consensus. Study characteristic information including the first author's name, publication year, sample size, study phase, treatment regime, tumor type, and other details of patients is listed in Table 1. Notably, studies used palbociclib combined with endocrine treatment were all randomized controlled trials, and studies adopted palbociclib as single agent were all non‐randomized controlled trials.

**Table 1:** Baseline characteristics

First author	Year	Phase	Histology	RB assessment (Biomarkers)	Treatment	Dose	Number	Age (median)	Gender M/F	Region
Cristofanilli M	2016	III	ER+, HER2‐, advanced BC	NR	Palbociclib‐Fulvestrant vs Placebo‐Fulvestrant	125 mg	521 (347/174)	57 (57/56)	0 + 521	17 countries
Finn RS	2016	II	ER+, HER2‐, advanced BC	NR	Palbociclib‐Letrozole vs Placebo‐Letrozole	125 mg	666 (444/222)	62/61	0 + 666	17 countries
Finn RS	2015	II	ER+, HER2‐, advanced BC	NR	Palbociclib‐Letrozole vs Letrozole	125 mg	165 (84/81)	63/64	0 + 165	USA
Tamura K	2016	I	ER+, HER2‐, advanced BC	NR	Palbociclib	125 mg	12	55 (24‐76)	0 + 12	Japan
DeMichele A	2014	II	Metastatic or Advanced BC	IHC (Antibody of MS‐107‐P, clone 1F8)	Palbociclib	125 mg	37	59 (39‐88)	0 + 37	USA
Dickson MA	2013	II	Advanced or metastatic WDLS/DDLS	IHC (RB [4H1] mouse monoclonal antibody)	Palbociclib	200 mg	30	65 (37‐83)	16 + 14	USA
Flaherty KT	2012	I	Advanced solid tumors	IHC	Palbociclib	Dose finding	41	54 (22‐77)	20 + 21	USA
Schwartz GK	2011	I	Rb‐positive advanced solid tumors or NHL	NR	Palbociclib	Dose finding 100/150/200/225 mg	33	63 (35‐78)	16 + 17	USA
Vaughn DJ	2015	II	Metastatic GCTs	IHC (RB1 mouse monoclonal antibody)	Palbociclib	125 mg	29	31 (17‐56)	26 + 4	USA

BC, breast cancer; GCTs, germ cell tumors; IHC, immunohistochemistry; NHL, non‐Hodgkin's lymphoma; NR, not report; WDLS/DDLS, well‐differentiated or dedifferentiated liposarcoma.

The clinical endpoints extracted from the trials were grade ≥3 and all‐grade (1‐5) adverse effects according to the National Cancer Institute (NCI) Common Toxicity Criteria version 3.0 or Common Terminology Criteria for Adverse Events (CTCAE) version 3.0. For safety endpoints, the types of different adverse events and total patients were extracted to calculate adverse event ratio with 95% CI in trials which used palbociclib as a single agent and OR with 95% CI in endocrine treatment‐combined trials. For efficacy endpoints, HR and 95% CI for PFS or OS were extracted following Parmar's method.

### 2.4 Statistical analysis

Statistical analysis of pooled PFS, OS, or toxicities was performed using the software Review manager 5.3 (Copenhagen, Sweden) or Comprehensive Meta‐Analysis (CMA) program 2 (Biostat, Englewood, NJ). The Cochrane Q statistic (significant at *P* < 0.10) and the *I* ^2^ value (significant heterogeneity if >50%) were used to examine heterogeneity.^12^ The pooled toxicities were analyzed using a fixed or random effects model, depending on heterogeneity. A fixed effects model was used when homogeneity was low (*P* > 0.10, *I* ^2^ ≤ 50%). A random effects model was adopted when there was a significant heterogeneity (*P* < 0.10, *I* ^2^ > 50%). The survival outcome effects of palbociclib were estimated by using forest plots of HR

## 3 RESULTS

### 3.1 Search results and study characteristics

**Figure 1:**
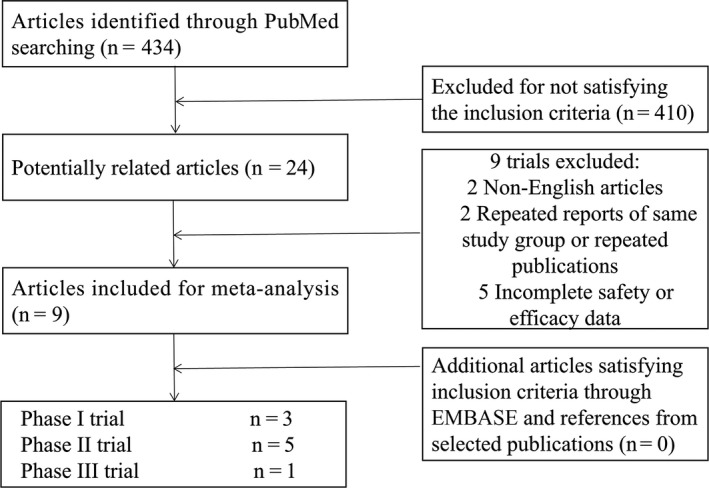
Flow diagram of the literature search and trial selection process

The initial search involved 434 potential studies, a total of 24 studies were identified as potentially relevant articles after title and abstract reviewing. The search of EMBASE publications did not supplement any additional results. After full‐text reviewing, fifteen studies were excluded due to lack of data on adverse effects or survival outcome. In total, 9 studies^8^, ^9^, ^10^, ^11^, ^13^, ^14^, ^15^, ^16^, ^17^ with 1534 patients which met our criteria were selected. The selection process is shown in Figure 1. Details of the nine eligible studies included in our final analysis are summarized in Table 1. These studies included three phase I, five phase II, and one phase III trials. Among these trials, six trials were designed of palbociclib as single agent, and three trials were designed of palbociclib plus other agent together as a therapy for patients. The eligible studies included four articles for ER‐positive and HER2‐negative breast cancer, one article for metastatic or advanced breast cancer, one article for metastatic germ cell tumor, one article for advanced or metastatic liposarcoma, and two articles for other Rb‐positive tumors.

### 3.2 Adverse effect

**Figure 2:**
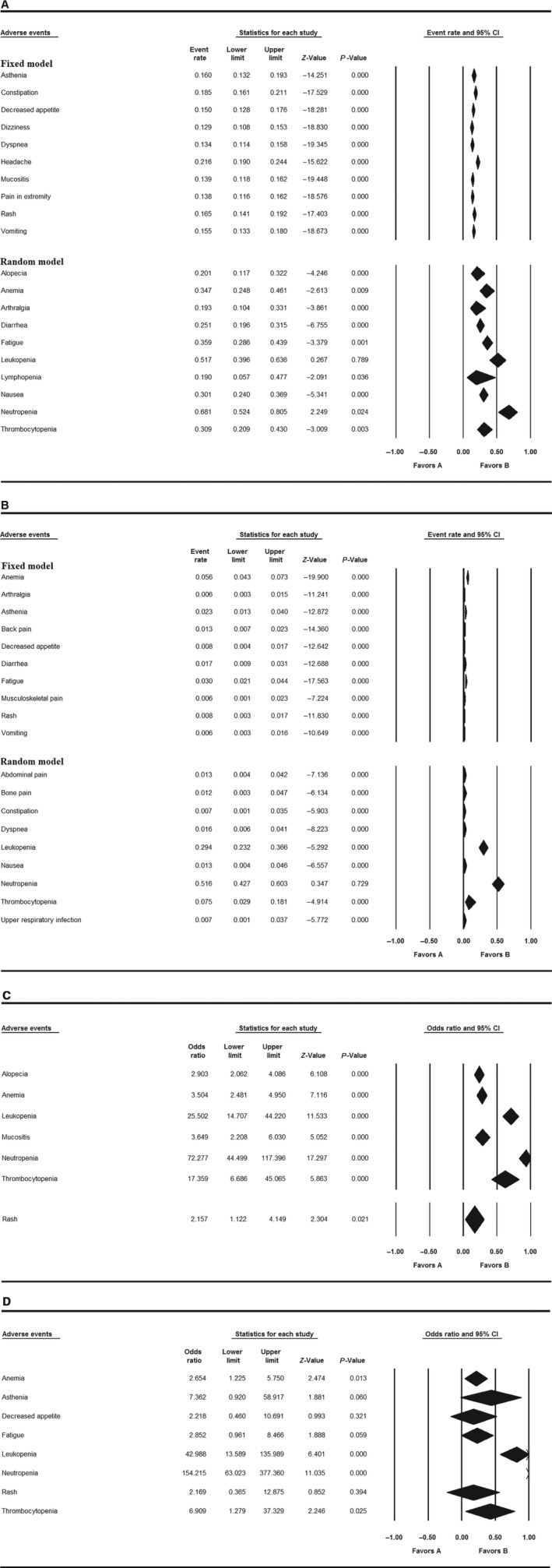
Adverse events and event rate/odds ratio with 95% CI. A, Top 10 all‐grade adverse events in single‐agent group; B, Top 10 grade ≥3 adverse events in single‐agent group; C, All‐grade adverse events with OR > 2 for endocrine treatment‐combined group; D, Grade ≥3 adverse events with OR > 2 for endocrine treatment‐combined group

Safety profiles were pooled together to analyze the risk factor for any side effects in the overall population with grade ≥3 or all‐grade adverse events (AEs). In all the single‐agent trials with all‐grade AEs analyzed with a fixed effects model, the highest risk was found for headache (event rate: 21.6%, 95% CI: 19.0%‐24.4%). In random model with all‐grade AEs of the single‐agent trials, neutropenia (68.1%, 95% CI: 52.4%‐80.5%), leukopenia (51.7%, 95% CI: 39.6%‐63.6%), fatigue (35.9%, 95% CI: 28.6%‐43.9%), anemia (34.7%, 95% CI: 24.8%‐46.1%), thrombocytopenia (30.9%, 95% CI: 20.9%‐43.0%), and nausea (30.1%, 95% CI 24.0%‐36.9%) were most common (Table 2A and Figure 2A).

**Table 2:** (A) Top 10 all‐grade adverse events for single‐agent group (B) Top 10 grade ≥3 adverse events for single‐agent group

Adverse events	Model	Event rate (%)	(95% CI) (%)	*Z*‐value	*P*‐value
			(Lower limit‐Upper limit)		
(A)					
Headache	Fixed	21.6	19.0‐24.4	−15.622	0
Constipation	Fixed	18.5	16.1‐21.1	−17.529	0
Rash	Fixed	16.5	14.1‐19.2	−17.403	0
Asthenia	Fixed	16	13.2‐19.3	−14.251	0
Vomiting	Fixed	15.5	13.3‐18.0	−18.673	0
Decreased appetite	Fixed	15	12.8‐17.6	−18.281	0
Mucositis	Fixed	13.9	11.8‐16.2	−19.448	0
Pain in extremity	Fixed	13.8	11.6‐16.2	−18.576	0
Dyspnea	Fixed	13.4	11.4‐15.8	−19.345	0
Dizziness	Fixed	12.9	10.8‐15.3	−18.83	0
Neutropenia	Random	68.1	52.4‐80.5	2.249	0.024
Leukopenia	Random	51.7	39.6‐63.6	0.267	0.789
Fatigue	Random	35.9	28.6‐43.9	−3.379	0.001
Anemia	Random	34.7	24.8‐46.1	−2.613	0.009
Thrombocytopenia	Random	30.9	20.9‐43.0	−3.009	0.003
Nausea	Random	30.1	24.0‐36.9	−5.341	0
Diarrhea	Random	25.1	19.6‐31.5	−6.755	0
Alopecia	Random	20.1	11.7‐32.2	−4.246	0
Arthralgia	Random	19.3	10.4‐33.1	−3.861	0
Lymphopenia	Random	19	5.7‐47.7	−2.091	0.036
(B)					
Anemia	Fixed	5.6	4.3‐7.3	−19.9	0
Fatigue	Fixed	3	2.1‐4.4	−17.563	0
Asthenia	Fixed	2.3	1.3‐4.0	−12.872	0
Diarrhea	Fixed	1.7	0.9‐3.1	−12.688	0
Back pain	Fixed	1.3	0.7‐2.3	−14.36	0
Decreased appetite	Fixed	0.8	0.4‐1.7	−12.642	0
Rash	Fixed	0.8	0.3‐1.7	−11.83	0
Arthralgia	Fixed	0.6	0.3‐1.5	−11.241	0
Musculoskeletal pain	Fixed	0.6	01‐2.3	−7.224	0
Vomiting	Fixed	0.6	0.3‐1.6	−10.649	0
Neutropenia	Random	51.6	42.7‐60.3	0.347	0.729
Leukopenia	Random	29.4	23.2‐36.6	−5.292	0
Thrombocytopenia	Random	7.5	2.9‐18.1	−4.914	0
Dyspnea	Random	1.6	0.6‐4.1	−8.223	0
Abdominal pain	Random	1.3	0.4‐4.2	−7.136	0
Nausea	Random	1.3	0.4‐4.6	−6.557	0
Bone pain	Random	1.2	0.3‐4.7	−6.134	0
Constipation	Random	0.7	0.1‐3.5	−5.903	0
Upper respiratory infection	Random	0.7	0.1‐3.7	−5.772	0

Serious TEAEs (grade ≥ 3) were pooled to reveal the clinical risk of palbociclib. Neutropenia (51.6%, 95% CI: 42.7%‐60.3%), leukopenia (29.4%, 95% CI: 23.2%‐36.6%), and thrombocytopenia (7.5%, 95% CI: 2.9%‐18.1%) were most common in decreasing order of frequency (Table 2B and Figure 2B).

In the analysis of all‐grade AEs of the 3 endocrine treatment‐combined trials,^9^, ^14^, ^15^ the odds ratio (OR) of neutropenia was 72.277 (95% CI: 44.499‐117.396). The OR of leukopenia was 25.502 (95% CI: 14.707‐44.220). The OR of thrombocytopenia was 17.359 (95% CI: 6.686‐45.065). The OR of mucositis was 3.649 (95% CI: 2.208‐6.030). The OR of anemia was 3.504 (95% CI: 2.481‐4.950). The OR of alopecia was 2.903 (95% CI: 2.062‐4.086). In serious TEAEs (grade ≥ 3), the OR of neutropenia was 154.215 (95% CI: 63.023‐377.360). The OR of leukopenia was 42.988 (95% CI: 13.589‐135.989). The OR of asthenia was 7.362 (95% CI: 0.920‐58.917). The OR of thrombocytopenia was 6.909 (95% CI: 1.279‐37.329). The OR of fatigue was 2.852 (95% CI: 0.961‐8.466). The OR of anemia was 2.654 (95% CI: 1.225‐5.750; Table 3A,B and Figure 2C,D).

**Table 3:** (A) All‐grade adverse events with OR > 2 for endocrine treatment‐combined group. (B) Grade ≥3 adverse events with OR > 2 for endocrine treatment‐combined group

Adverse events	Model	Odds Ratio	95% CI	*Z*‐value	*P*‐value
			(Lower limit‐Upper limit)		
(A)					
Neutropenia	Fixed	72.28	44.499‐117.396	17.297	0
Leukopenia	Fixed	25.5	14.707‐44.220	11.533	0
Thrombocytopenia	Fixed	17.36	6.686‐45.065	5.863	0
Mucositis	Fixed	3.649	2.208‐6.030	5.052	0
Anemia	Fixed	3.504	2.481‐4.950	7.116	0
Alopecia	Fixed	2.903	2.062‐4.086	6.108	0
Rash	Random	2.157	1.122‐4.149	2.304	0.021
(B)					
Neutropenia	Fixed	154.2	63.023‐377.360	11.035	0
Leukopenia	Fixed	42.99	13.589‐135.989	6.401	0
Asthenia	Fixed	7.362	0.920‐58.917	1.881	0.06
Thrombocytopenia	Fixed	6.909	1.279‐37.329	2.246	0.025
Fatigue	Fixed	2.852	0.961‐8.466	1.888	0.059
Anemia	Fixed	2.654	1.225‐5.750	2.474	0.013
Decreased appetite	Fixed	2.218	0.460‐10.691	0.993	0.321
Rash	Fixed	2.169	0.365‐12.875	0.852	0.394

**Figure 3:**
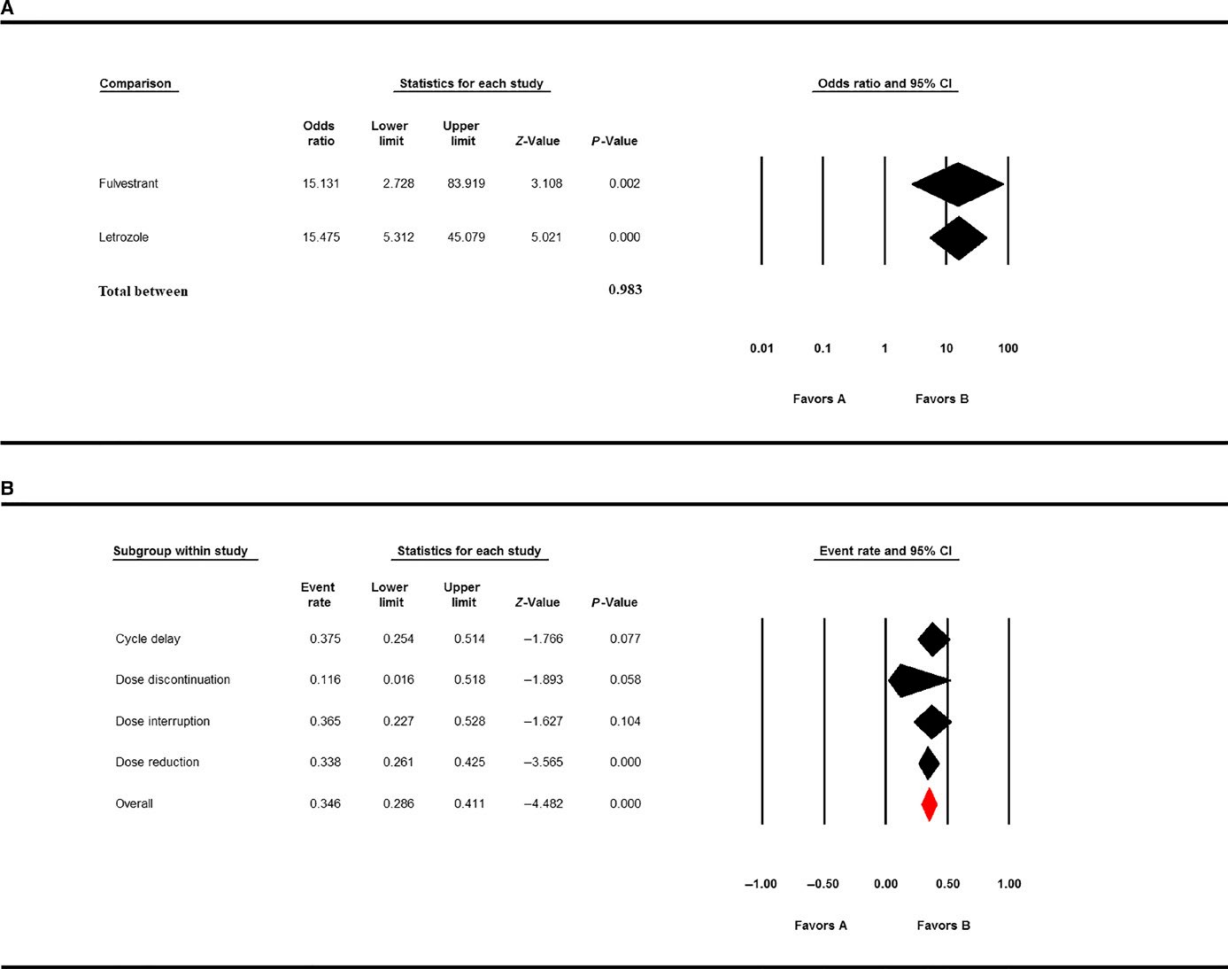
A, Comparison of adverse events in fulvestrant‐palbociclib group and letrozole‐palbociclib group. B, Unexpected treatment changes due to side effects

Between the two endocrine treatment‐combined groups, the OR of hematologic adverse events of fulvestrant‐combined group was 15.131 (95% CI: 2.728‐83.919), and the OR of hematologic adverse events of letrozole‐combined group was 15.475 (95% CI: 5.312‐45.079), but the difference between the two groups was statistically insignificant (*P* = 0.983; Figure 3A).

Serious adverse effects might affect treatment outcome; therefore, we analyzed the incidence of unexpected treatment changes due to side effects. Cycle delay was the most common events due to adverse effects (event rate: 37.5%, 95% CI: 25.4%‐51.4%). The event rate of dose interruption due to side effects was 36.5% (95% CI: 22.7%‐52.8%). The event rate of dose reduction due to adverse effects was 33.8% (95% CI: 26.1%‐42.5%). The event rate of dose discontinuation due to side effects was 11.6% (95% CI: 1.6%‐51.8%; Figure 3B).

Details including adverse events, study names, and statistical results are shown in Figures S1a, S1b, S2a, S2b, S3a, S3b, S4a, and S4b.

### 3.3 OS and PFS

**Figure 4:**
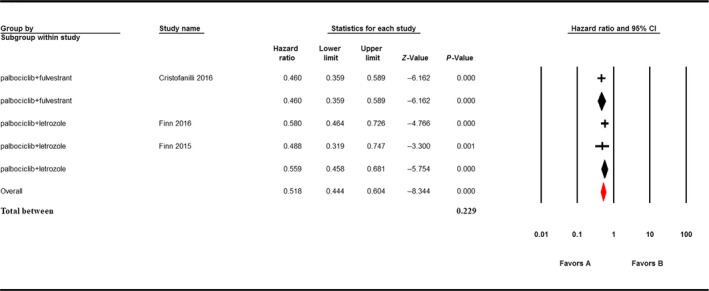
The HR and 95% CI for PFS in endocrine treatment‐combined group

The PFS analysis was based on three endocrine treatment‐combined trials and four single‐agent trials, including 1464 patients. In endocrine treatment‐combined studies, our analysis showed that the utility of palbociclib in treatment was beneficial in prolonging PFS (HR: 0.518, 95% CI: 0.444‐0.604). Subgroup analysis demonstrated that the utility of palbociclib combined with fulvestrant (HR: 0.460, 95% CI: 0.359‐0.589) was more beneficial than the utility of palbociclib combined with letrozole (HR: 0.559, 95% CI: 0.458‐0.681) in prolonging PFS; however, the difference was statistically insignificant (*P* = 0.229; Figure 4). Within the three endocrine treatment‐combined trials for breast cancer, Cristofanilli et al^9^ showed the median PFS was 9.5 months (95% CI: 9.2‐11.0) in the fulvestrant plus palbociclib group and 4.6 months (95% CI: 3.5‐5.6) in the fulvestrant plus placebo group (HR: 0.46, 95% CI: 0.36‐0.59, *P* < 0.0001). Finn et al^15^ reported the median PFS was 24.8 months (95% CI: 22.1—not estimable) in the palbociclib‐letrozole group, as compared with 14.5 months (95% CI: 12.9‐17.1) in the placebo‐letrozole group (HR: 0.58, 95% CI: 0.46‐0.72, *P* < 0.001). Finn et al^14^ manifested the median PFS was 10.2 months in the letrozole group (95% CI: 5.7‐12.6) and 20.2 months in the palbociclib‐letrozole group (95% CI: 13.8‐27.5; HR: 0.488, 95% CI: 0.319‐0.748; one‐sided *P* < 0.10).

In single‐agent studies, one phase II trial^8^ for well‐differentiated or dedifferentiated liposarcoma showed the median PFS was 4.5 months, which was 3.7 months in the phase II trial^13^ for Rb‐positive advanced breast cancer. Regarding the study^11^ for germ cell tumors, the median PFS for all evaluable patients who received treatment was 2.7 months. One phase I dose‐finding trial^17^ for solid tumor or NHL reported the duration of PFS ranged from 28 to 280 days. Only one study^14^ assessed OS. Median OS was 37.5 months in the palbociclib‐letrozole group (95% CI: 28.4—not estimable; 30 events) and 33.3 months in the letrozole group (95% CI: 26.4—not estimable; 31 events; HR: 0.813, 95% CI: 0.492‐1.345; two‐sided *P* = 0.317).

### 3.4 Quality assessment

**Figure 5:**
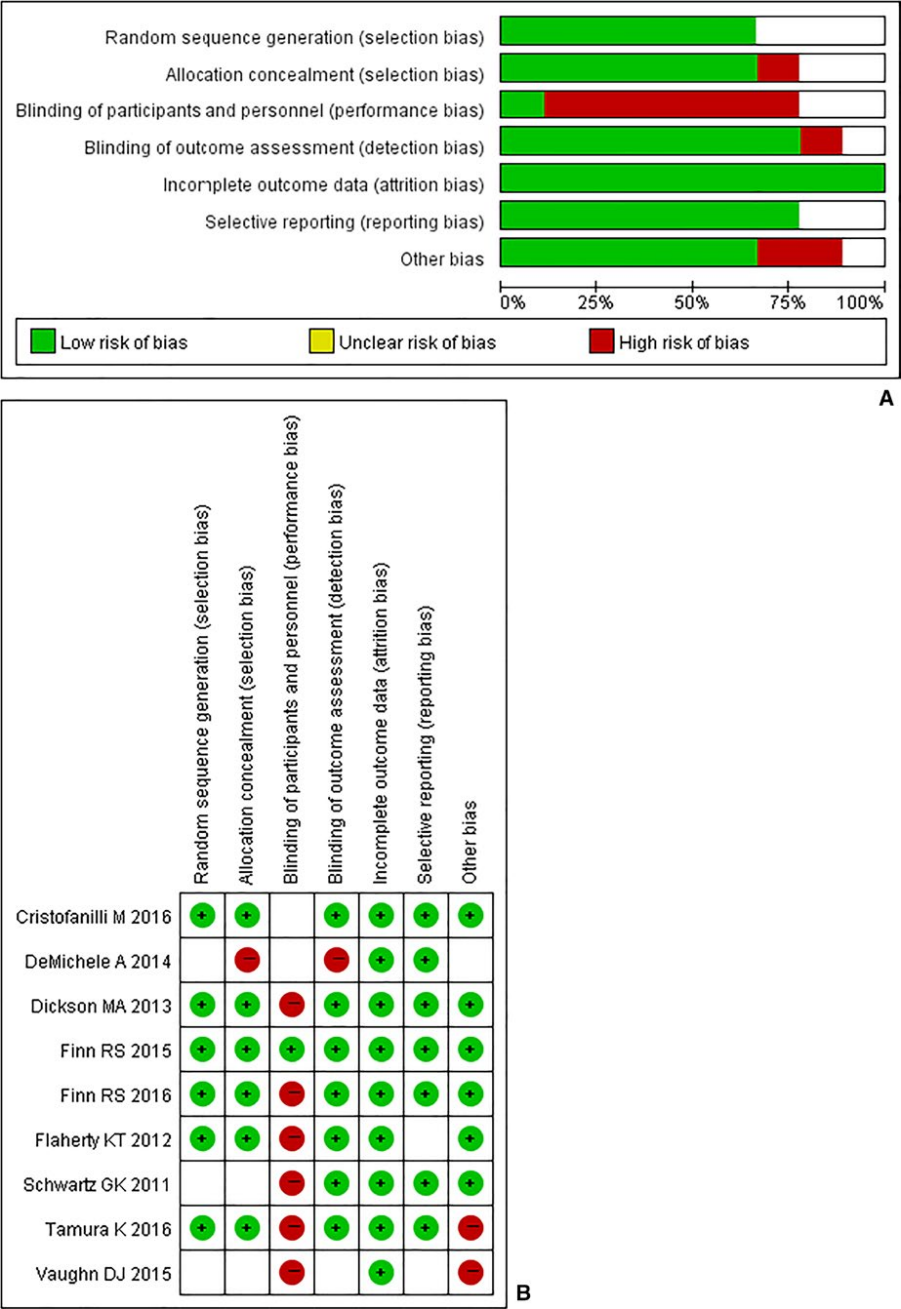
Quality assessment. A, Risk of bias graph; B, risk of bias summary

Review Manager 5.3 (Copenhagen, Sweden) was used to measure quality assessment. QUADAS‐2^18^ was used to estimate the quality of eligible studies. Overall, the quality of the studies was satisfactory. The results are shown in Figure 5A,B.

## 4 DISCUSSION

Our analysis pooled data from nine clinical trials involving 1534 patients. Based on safety and efficacy analysis, we provided evidence regarding the beneficial effect of palbociclib in ER‐positive and HER2‐negative breast cancer and the potential value of palbociclib in Rb‐positive germ cell tumors and Rb‐positive liposarcoma.

Serious adverse effect due to poor specificity usually restricts potential therapeutic value in oncology.^19^ Our results suggested there were more adverse effects in the group that used palbociclib‐containing regimen but all remained within expected parameters, at the same time, toxicities were relatively manageable. In the single‐agent trials, the most frequently observed all‐grade adverse events in patients treated with palbociclib were neutropenia, leukopenia, fatigue, anemia, thrombocytopenia, and nausea. The most common grade 3 or more toxicities were neutropenia, leukopenia, and thrombocytopenia. OR > 2 was considered as a criterion of strong clinical value.^20^ According to the OR value, within the three endocrine treatment‐combined trials, neutropenia had a higher occurrence in palbociclib regimen patients. The adverse events of palbociclib are CDK 4/6 inhibitor relevant, because palbociclib inhibits an upstream target of cell progression from G1 phase to S phase without specificity,^21^ which suggested that patients who were administered palbociclib should be strictly monitored and managed with preventive drugs or dose reduction.

Hematologic adverse events had high occurrence in the palbociclib group. The underlying mechanism of palbociclib‐related hematologic toxicities is possibly associated with myelosuppression, which may result from an effect of CDK 4/6 on‐target inhibition.^14^ On‐target refers to adverse effects caused by exaggerated and adverse pharmacologic effects of the target of interest.^22^ Currently, bone marrow hematopoietic stem and progenitor cells (HSPCs) have been found to require the activity of CDK 4/6 for proliferation.^23^ Due to poor sensitivity of this CDK 4/6 inhibitor, it resulted in the inhibition of HSPCs and caused hematologic adverse events. In addition, myelosuppression induced by CDK 4/6 inhibitor often causes dose reduction and therefore affects efficacy.^19^ Our results also demonstrated that the most common events due to side effects were cycle delay, dose interruption, and dose reduction, which might have effect on efficacy. To minimize myelosuppressive effects and related complications, current therapeutic approaches are depended on growth factors which are suboptimal^19^ and the US Food and Drug Administration has approved drugs for antitumor therapy‐induced myelosuppression such as filgrastim for neutropenia.^24^, ^25^, ^26^, ^27^, ^28^, ^29^, ^30^

In terms of efficacy, the addition of palbociclib in treatment regimen conferred a progression‐free benefit. Since CDK 4/6 can promote cell‐cycle entry from the G1 phase to the S phase by phosphorylating Rb protein, palbociclib inhibits CDK 4/CDK 6 therefore leading to tumor growth limitation.^5^, ^31^ In particular, the CDK 4/6 inhibitor palbociclib has shown high activity in ER‐positive and HER2‐negative advanced breast cancer, and it may result from the inhibition of CDK 4/6. Cyclin D1 is essential for breast cancer formation by coupling with CDK 4/6 to promote cell cycling. Binding to ER‐alpha subunit drives cyclin D1 transcription. By inhibiting CDK 4/6, palbociclib has a positive effect on the treatment of ER‐positive and HER2‐negative advanced breast cancer.^32^, ^33^ Our analysis showed that palbociclib is beneficial in prolonging PFS (HR: 0.518, 95% CI: 0.444‐0.604) in ER‐positive and HER2‐negative breast cancer patients. Three randomized controlled trials^9^, ^14^, ^15^ all demonstrated people could get the clinical benefit from palbociclib. One double‐blind, phase III trial included breast cancer patients that had relapsed or progressed during prior endocrine therapy. The result showed a prolonged median PFS from 4.6 months (placebo‐fulvestrant group) to 9.5 months (palbociclib 125 mg oral daily‐fulvestrant group; HR: 0.46 95% CI: 0.36‐0.59).^9^ In addition, in another phase II, multicenter open‐label randomized study,^14^ the result demonstrated that the median PFS was 20.2 months in the palbociclib‐letrozole group (125 mg daily) compared with 10.2 months in the letrozole group (HR: 0.488, 95% CI: 0.319‐0.748) and a phase III study^9^ showed that the median PFS increased from 14.5 months in the placebo‐letrozole group to 24.8 months in the palbociclib‐letrozole group (125 mg daily; HR: 0.58, 95% CI: 0.46‐0.72). The difference in median PFS within these RCTs might be related to different patient samples, pretreatment diseases, and treatment regimens. A phase II, single arm trial^13^ of palbociclib also demonstrated the single agent was well tolerated and active in patients with hormone receptor‐positive and Rb‐positive breast cancer. Finally, our estimation about OS did not manifest in statistical significance (*P* > 0.10).^14^ All above pooled results proved that palbociclib had high clinical value in the treatment for ER‐positive and HER2‐negative breast cancer, but the therapeutic value of palbociclib in other tumors is unclear.

Endocrine therapy has played the leading role in the treatment for ER‐positive breast cancer.^34^ Notably, our subgroup analysis revealed that there was no statistically significant difference in hematologic side effects and efficacy between fulvestrant‐palbociclib group and letrozole‐palbociclib group, which might be due to the small number of studies. Nowadays, more and more women have gained resistance to endocrine therapy, resulting a relapse of breast cancer.^35^ Results from our study supported the scientific evidence that palbociclib had high activity in ER‐positive and HER2‐negative breast cancer lines. In addition, the finding in one phase II^14^ and one phase III trial^9^ suggested that palbociclib is active in both patients who have acquired resistance to endocrine therapy and who have not received such therapy, when combined with endocrine therapy.

Remarkably, one phase I trial^10^ suggested the maximum tolerated dose and recommended phase II dose of palbociclib were 125 mg once daily, at which dose neutropenia was the sole significant toxicity. It should be pointed out that six^9^, ^11^, ^13^, ^14^, ^15^, ^17^ included trials adopted 125 mg palbociclib daily for patients. This dose of palbociclib might be associated with its relatively better survival outcome and lower toxicity.

Our analysis was limited by the small sample size and absence of blinding. Since palbociclib is a relatively new drug, trials about it are few, especially phase III trials. And only one study provided the OS data, so prolonged follow‐ups are essential. The previous study reported a strong association between PFS and QoL among cancer patients.^36^ Unfortunately, we did not analyze data concerning QoL because they were lacking or not homogeneous. More randomized controlled trials, OS data, and QoL included trials are needed to further validate our results.

In conclusion, our study showed that palbociclib has high activity in ER‐positive and HER2‐negative breast cancer and prolonged PFS in Rb‐positive tumors. In terms of adverse effect, hematologic adverse events were common, which suggested preventive measures should be adopted to reduce toxicity. More studies are needed to better understand the long‐term efficacy and toxicity of palbociclib.

## CONFLICT OF INTEREST

None declared.
