# Supplementary material for: Safety and efficacy profile of cyclin‐dependent kinases 4/6 inhibitor palbociclib in cancer therapy: A meta‐analysis of clinical trials
Source: Cancer Med. 2019 Mar 21;8(4):1389–400. doi: 10.1002/cam4.1970 (PMC6488107; doi:10.1002/cam4.1970)

Safety and Efficacy Profile of CDK 4/6 Inhibitor Palbociclib in Cancer Therapy: A Meta-Analysis of Clinical Trials

Linghong Guo2*, Yuanyuan Hu2*, Xi Chen3, Qingfang Li1, Benling Wei4, Xuelei Ma1*
1State Key Laboratory of Biotherapy and Cancer Center, West China Hospital, Sichuan University, Chengdu, PR China
2Weat China School of Medicine, Sichuan University, Chengdu, PR China
3Weat China School of Stomatology, Sichuan University, Chengdu, PR China
4General Hospital of Xuzhou Mining Group, Xuzhou, PR China
*Note: Linghong Guo, Yuanyuan Hu and Xuelei Ma contributed equally to this work
*Corresponding author: Xuelei Ma, West China Hospital, No. 37, Guoxue Alley, Chengdu 610041, PR China. Tel: +86-28-85475576, Fax: +86-28-85475576, E-mail: drmaxuelei@gmail.com

Figure legend
Supplementary Figure S1a. All grade adverse events in single arm groups (Fixed model).
Supplementary Figure S1b. All grade adverse events in single arm groups (Random model).
Supplementary Figure S2a. Grade≥3 adverse events in single arm groups (Fixed model).
Supplementary Figure S2b. Grade≥3 adverse events in single arm groups (Random model).
Supplementary Figure S3a. All grade adverse events in controlled groups (Fixed model).
Supplementary Figure S3b. All grade adverse events in controlled groups (Random model).
Supplementary Figure S4a. Grade≥3 adverse events in controlled groups (Fixed model).
Supplementary Figure S4b. Grade≥3 adverse events in controlled groups (Random model).

Supplementary Figure S1a. All grade adverse events in single arm groups (Fixed model).

Group by Subgroup within study	Study name	Subgroup within study	Event rate	Lower limit	Upper limit	Z-Value	p-Value
Asthenia	Schwartz GK 2011	Asthenia	0.061	0.015	0.212	-3.757	0.000
Asthenia	Finn RS 2016	Asthenia	0.169	0.137	0.207	-12.579	0.000
Asthenia	Finn RS 2015	Asthenia	0.133	0.075	0.224	-5.804	0.000
Asthenia			0.160	0.132	0.193	-14.251	0.000
Constipation	DeMichele A 2014	Constipation	0.081	0.026	0.223	-4.031	0.000
Constipation	Schwartz GK 2011	Constipation	0.212	0.105	0.383	-3.082	0.002
Constipation	Cristofanilli M 2016	Constipation	0.191	0.153	0.236	-10.532	0.000
Constipation	Finn RS 2016	Constipation	0.194	0.160	0.233	-11.876	0.000
Constipation	Finn RS 2015	Constipation	0.120	0.066	0.210	-5.895	0.000
Constipation			0.185	0.161	0.211	-17.529	0.000
Decreased appetite	Cristofanilli M 2016	Decreased appetite	0.151	0.117	0.192	-11.490	0.000
Decreased appetite	Finn RS 2016	Decreased appetite	0.149	0.119	0.185	-13.082	0.000
Decreased appetite	Finn RS 2015	Decreased appetite	0.157	0.093	0.251	-5.575	0.000
Decreased appetite			0.150	0.128	0.176	-18.281	0.000
Dizziness	Cristofanilli M 2016	Dizziness	0.119	0.089	0.157	-12.042	0.000
Dizziness	Finn RS 2016	Dizziness	0.142	0.112	0.178	-13.232	0.000
Dizziness	Finn RS 2015	Dizziness	0.096	0.049	0.181	-6.017	0.000
Dizziness			0.129	0.108	0.153	-18.830	0.000
Dysgeusia	DeMichele A 2014	Dysguesia	0.081	0.026	0.223	-4.031	0.000
Dysgeusia	Finn RS 2016	Dysguesia	0.101	0.077	0.133	-13.878	0.000
Dysgeusia			0.100	0.076	0.130	-14.446	0.000
Dysphasia	Flaherty KT 2012	Dysphasia	0.024	0.003	0.154	-3.644	0.000
Dysphasia	Finn RS 2016	Dysphasia	0.092	0.069	0.123	-13.941	0.000
Dysphasia			0.089	0.067	0.119	-14.345	0.000
Dyspnea	Flaherty KT 2012	Dyspnea	0.049	0.012	0.175	-4.097	0.000
Dyspnea	Schwartz GK 2011	Dyspnea	0.121	0.046	0.282	-3.714	0.000
Dyspnea	Cristofanilli M 2016	Dyspnea	0.116	0.086	0.154	-12.080	0.000
Dyspnea	Finn RS 2016	Dyspnea	0.149	0.119	0.185	-13.082	0.000
Dyspnea	Finn RS 2015	Dyspnea	0.157	0.093	0.251	-5.575	0.000
Dyspnea			0.134	0.114	0.158	-19.345	0.000
Epistaxis	Schwartz GK 2011	Epistaxis	0.121	0.046	0.282	-3.714	0.000
Epistaxis	Finn RS 2015	Epistaxis	0.108	0.057	0.195	-5.968	0.000
Epistaxis			0.112	0.066	0.184	-7.027	0.000
Headache	Cristofanilli M 2016	Headache	0.232	0.190	0.279	-9.389	0.000
Headache	Finn RS 2016	Headache	0.214	0.178	0.255	-11.244	0.000
Headache	Finn RS 2015	Headache	0.145	0.084	0.238	-5.696	0.000
Headache			0.216	0.190	0.244	-15.622	0.000
Mucositis	DeMichele A 2014	Mucositis	0.189	0.093	0.347	-3.467	0.001
Mucositis	Schwartz GK 2011	Mucositis	0.091	0.030	0.247	-3.803	0.000
Mucositis	Vaughn DJ 2015	Mucositis	0.034	0.005	0.208	-3.274	0.001
Mucositis	Cristofanilli M 2016	Mucositis	0.125	0.094	0.164	-11.959	0.000
Mucositis	Finn RS 2016	Mucositis	0.153	0.123	0.190	-12.977	0.000
Mucositis	Finn RS 2015	Mucositis	0.120	0.066	0.210	-5.895	0.000
Mucositis			0.139	0.118	0.162	-19.448	0.000
Myalgia	DeMichele A 2014	Myalgia	0.135	0.057	0.286	-3.860	0.000
Myalgia	Finn RS 2016	Myalgia	0.119	0.092	0.153	-13.653	0.000
Myalgia	Finn RS 2015	Myalgia	0.108	0.057	0.195	-5.968	0.000
Myalgia			0.119	0.095	0.148	-15.386	0.000
Pain in extremity	Cristofanilli M 2016	Pain in extremity	0.125	0.094	0.164	-11.959	0.000
Pain in extremity	Finn RS 2016	Pain in extremity	0.153	0.123	0.190	-12.977	0.000
Pain in extremity	Finn RS 2015	Pain in extremity	0.096	0.049	0.181	-6.017	0.000
Pain in extremity			0.138	0.116	0.162	-18.576	0.000
Peripheral neuropathy	Flaherty KT 2012	Peripheral neuropathy	0.024	0.003	0.154	-3.644	0.000
Peripheral neuropathy	Finn RS 2015	Peripheral neuropathy	0.096	0.049	0.181	-6.017	0.000
Peripheral neuropathy			0.082	0.043	0.151	-6.905	0.000
Pleural effusion	Flaherty KT 2012	Pleural effusion	0.024	0.003	0.154	-3.644	0.000
Pleural effusion	Cristofanilli M 2016	Pleural effusion	0.017	0.008	0.038	-9.796	0.000
Pleural effusion			0.018	0.009	0.038	-10.446	0.000
Pyrexia	Schwartz GK 2011	Pyrexia	0.091	0.030	0.247	-3.803	0.000
Pyrexia	Cristofanilli M 2016	Pyrexia	0.110	0.081	0.148	-12.149	0.000
Pyrexia	Finn RS 2016	Pyrexia	0.124	0.096	0.158	-13.580	0.000
Pyrexia			0.117	0.097	0.141	-18.598	0.000
Rash	DeMichele A 2014	Rash	0.027	0.004	0.168	-3.535	0.000
Rash	Schwartz GK 2011	Rash	0.182	0.084	0.350	-3.333	0.001
Rash	Cristofanilli M 2016	Rash	0.151	0.117	0.192	-11.490	0.000
Rash	Finn RS 2016	Rash	0.178	0.145	0.216	-12.334	0.000
Rash			0.165	0.141	0.192	-17.403	0.000
Vomiting	DeMichele A 2014	Vomiting	0.054	0.014	0.192	-3.937	0.000
Vomiting	Schwartz GK 2011	Vomiting	0.091	0.030	0.247	-3.803	0.000
Vomiting	Cristofanilli M 2016	Vomiting	0.168	0.132	0.211	-11.107	0.000
Vomiting	Finn RS 2016	Vomiting	0.155	0.125	0.192	-12.923	0.000
Vomiting	Finn RS 2015	Vomiting	0.145	0.084	0.238	-5.696	0.000
Vomiting			0.155	0.133	0.180	-18.673	0.000
Weight loss	DeMichele A 2014	Weight loss	0.027	0.004	0.168	-3.535	0.000
Weight loss	Vaughn DJ 2015	Weight loss	0.034	0.005	0.208	-3.274	0.001
Weight loss			0.031	0.008	0.114	-4.815	0.000

Event rate and 95% CI

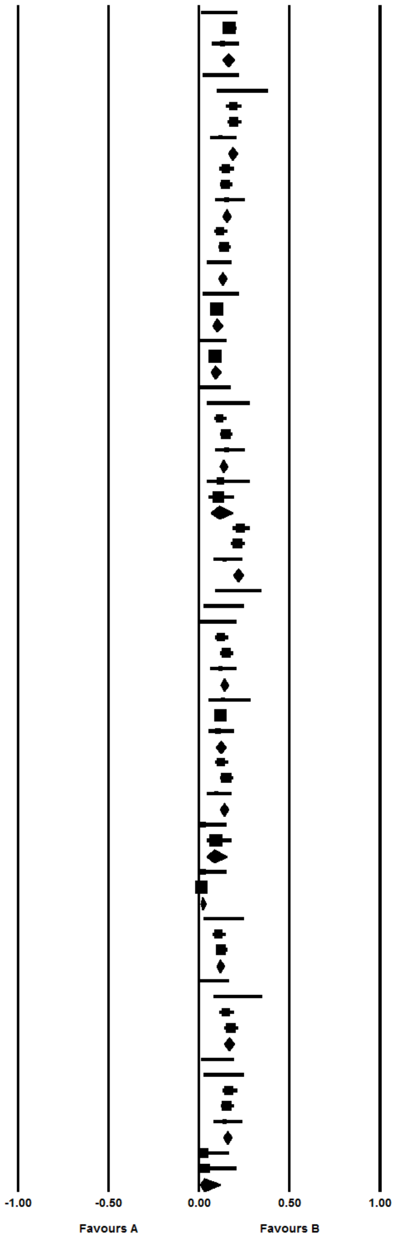

Supplementary Figure S1b. All grade adverse events in single arm groups (Random model).

Group by / Subgroup within study	Study name		Event rate	Lower limit	Upper limit	Z-Value	p-Value
			Statistics for each study				
Abdominal pain	Cristofanilli M 2016	Abdominal pain	0.078	0.054	0.112	-12.303	0.000
Abdominal pain	Finn RS 2016	Abdominal pain	0.113	0.086	0.146	-13.751	0.000
Abdominal pain			0.096	0.067	0.136	-11.231	0.000
Alopecia	Schwartz GK 2011	Alopecia	0.061	0.015	0.212	-3.757	0.000
Alopecia	Cristofanilli M 2016	Alopecia	0.168	0.132	0.211	-11.107	0.000
Alopecia	Finn RS 2016	Alopecia	0.329	0.287	0.374	-7.063	0.000
Alopecia	Finn RS 2015	Alopecia	0.217	0.141	0.318	-4.821	0.000
Alopecia			0.201	0.117	0.322	-4.246	0.000
Anemia	DeMichele A 2014	Anemia	0.649	0.485	0.784	1.780	0.075
Anemia	Flaherty KT 2012	Anemia	0.098	0.037	0.233	-4.227	0.000
Anemia	Schwartz GK 2011	Anemia	0.636	0.463	0.781	1.546	0.122
Anemia	Vaughn DJ 2015	Anemia	0.310	0.170	0.497	-1.989	0.047
Anemia	Cristofanilli M 2016	Anemia	0.278	0.234	0.328	-7.934	0.000
Anemia	Finn RS 2016	Anemia	0.241	0.203	0.283	-10.339	0.000
Anemia	Finn RS 2015	Anemia	0.349	0.255	0.457	-2.700	0.007
Anemia			0.347	0.248	0.461	-2.613	0.009
Anorexia	DeMichele A 2014	Anorexia	0.270	0.152	0.433	-2.683	0.007
Anorexia	Schwartz GK 2011	Anorexia	0.121	0.046	0.282	-3.714	0.000
Anorexia	Vaughn DJ 2015	Anorexia	0.069	0.017	0.238	-3.552	0.000
Anorexia			0.153	0.066	0.316	-3.570	0.000
Arthralgia	DeMichele A 2014	Arthralgia	0.081	0.026	0.223	-4.031	0.000
Arthralgia	Cristofanilli M 2016	Arthralgia	0.142	0.109	0.183	-11.661	0.000
Arthralgia	Finn RS 2016	Arthralgia	0.333	0.291	0.379	-6.885	0.000
Arthralgia	Finn RS 2015	Arthralgia	0.229	0.151	0.331	-4.648	0.000
Arthralgia			0.193	0.104	0.331	-3.861	0.000
Back pain	Cristofanilli M 2016	Back pain	0.148	0.114	0.189	-11.548	0.000
Back pain	Finn RS 2016	Back pain	0.216	0.180	0.257	-11.171	0.000
Back pain	Finn RS 2015	Back pain	0.145	0.084	0.238	-5.696	0.000
Back pain			0.173	0.127	0.232	-8.365	0.000
Cough	Schwartz GK 2011	Cough	0.061	0.015	0.212	-3.757	0.000
Cough	Cristofanilli M 2016	Cough	0.148	0.114	0.189	-11.548	0.000
Cough	Finn RS 2016	Cough	0.250	0.212	0.292	-10.024	0.000
Cough	Finn RS 2015	Cough	0.120	0.066	0.210	-5.895	0.000
Cough			0.156	0.096	0.243	-5.988	0.000
Diarrhea	DeMichele A 2014	Diarrhea	0.162	0.075	0.317	-3.682	0.000
Diarrhea	Schwartz GK 2011	Diarrhea	0.485	0.322	0.651	-0.174	0.862
Diarrhea	Tamura K 2016	Diarrhea	0.250	0.083	0.552	-1.648	0.099
Diarrhea	Cristofanilli M 2016	Diarrhea	0.214	0.174	0.261	-9.897	0.000
Diarrhea	Finn RS 2016	Diarrhea	0.261	0.223	0.304	-9.622	0.000
Diarrhea	Finn RS 2015	Diarrhea	0.205	0.131	0.305	-4.987	0.000
Diarrhea			0.251	0.196	0.315	-6.755	0.000
Fatigue	DeMichele A 2014	Fatigue	0.405	0.261	0.568	-1.144	0.253
Fatigue	Flaherty KT 2012	Fatigue	0.073	0.024	0.204	-4.234	0.000
Fatigue	Schwartz GK 2011	Fatigue	0.606	0.434	0.756	1.209	0.227
Fatigue	Vaughn DJ 2015	Fatigue	0.103	0.034	0.276	-3.542	0.000
Fatigue	Tamura K 2016	Fatigue	0.333	0.131	0.624	-1.132	0.258
Fatigue	Cristofanilli M 2016	Fatigue	0.391	0.341	0.444	-4.005	0.000
Fatigue	Finn RS 2016	Fatigue	0.374	0.330	0.420	-5.257	0.000
Fatigue	Finn RS 2015	Fatigue	0.410	0.309	0.518	-1.637	0.102
Fatigue			0.359	0.286	0.439	-3.379	0.001
Hot flushes	Cristofanilli M 2016	Hot flushes	0.154	0.119	0.196	-11.429	0.000
Hot flushes	Finn RS 2016	Hot flushes	0.209	0.174	0.250	-11.388	0.000
Hot flushes	Finn RS 2015	Hot flushes	0.205	0.131	0.305	-4.987	0.000
Hot flushes			0.186	0.149	0.231	-10.703	0.000
Insomnia	Cristofanilli M 2016	Insomnia	0.096	0.069	0.132	-12.272	0.000
Insomnia	Finn RS 2016	Insomnia	0.149	0.119	0.185	-13.082	0.000
Insomnia			0.121	0.078	0.184	-7.916	0.000
Leukopenia	Dickson MA 2013	Leukopenia	0.931	0.762	0.983	3.552	0.000
Leukopenia	Flaherty KT 2012	Leukopenia	0.122	0.052	0.261	-4.136	0.000
Leukopenia	Schwartz GK 2011	Leukopenia	0.667	0.492	0.805	1.877	0.061
Leukopenia	Vaughn DJ 2015	Leukopenia	0.759	0.573	0.880	2.639	0.008
Leukopenia	Cristofanilli M 2016	Leukopenia	0.496	0.443	0.548	-0.162	0.872
Leukopenia	Finn RS 2016	Leukopenia	0.390	0.345	0.436	-4.612	0.000
Leukopenia	Finn RS 2015	Leukopenia	0.434	0.332	0.542	-1.204	0.229
Leukopenia			0.517	0.396	0.636	0.267	0.789
Lymphopenia	DeMichele A 2014	Lymphopenia	0.351	0.216	0.515	-1.780	0.075
Lymphopenia	Dickson MA 2013	Lymphopenia	0.414	0.252	0.596	-0.924	0.356
Lymphopenia	Schwartz GK 2011	Lymphopenia	0.242	0.126	0.415	-2.805	0.005
Lymphopenia	Tamura K 2016	Lymphopenia	0.250	0.083	0.552	-1.648	0.099
Lymphopenia	Cristofanilli M 2016	Lymphopenia	0.017	0.008	0.038	-9.796	0.000
Lymphopenia			0.190	0.057	0.477	-2.091	0.036
Nausea	DeMichele A 2014	Nausea	0.243	0.132	0.405	-2.962	0.003
Nausea	Flaherty KT 2012	Nausea	0.049	0.012	0.175	-4.097	0.000
Nausea	Schwartz GK 2011	Nausea	0.424	0.270	0.595	-0.867	0.386
Nausea	Cristofanilli M 2016	Nausea	0.325	0.277	0.376	-6.371	0.000
Nausea	Finn RS 2016	Nausea	0.351	0.308	0.397	-6.167	0.000
Nausea	Finn RS 2015	Nausea	0.253	0.171	0.357	-4.288	0.000
Nausea			0.301	0.240	0.369	-5.341	0.000
Neutropenia	DeMichele A 2014	Neutropenia	0.405	0.261	0.568	-1.144	0.253
Neutropenia	Dickson MA 2013	Neutropenia	0.966	0.792	0.995	3.274	0.001
Neutropenia	Flaherty KT 2012	Neutropenia	0.317	0.194	0.473	-2.286	0.022
Neutropenia	Schwartz GK 2011	Neutropenia	0.424	0.270	0.595	-0.867	0.386
Neutropenia	Vaughn DJ 2015	Neutropenia	0.793	0.610	0.904	2.931	0.003
Neutropenia	Tamura K 2016	Neutropenia	0.833	0.523	0.958	2.078	0.038
Neutropenia	Cristofanilli M 2016	Neutropenia	0.809	0.764	0.847	10.532	0.000
Neutropenia	Finn RS 2016	Neutropenia	0.795	0.755	0.830	11.531	0.000
Neutropenia			0.681	0.524	0.805	2.249	0.024
Thrombocytopenia	DeMichele A 2014	Thrombocytopenia	0.568	0.406	0.716	0.819	0.413
Thrombocytopenia	Dickson MA 2013	Thrombocytopenia	0.379	0.224	0.564	-1.287	0.198
Thrombocytopenia	Schwartz GK 2011	Thrombocytopenia	0.576	0.405	0.730	0.867	0.386
Thrombocytopenia	Vaughn DJ 2015	Thrombocytopenia	0.241	0.120	0.427	-2.639	0.008
Thrombocytopenia	Tamura K 2016	Thrombocytopenia	0.417	0.185	0.692	-0.575	0.566
Thrombocytopenia	Cristofanilli M 2016	Thrombocytopenia	0.212	0.172	0.258	-9.979	0.000
Thrombocytopenia	Finn RS 2016	Thrombocytopenia	0.155	0.125	0.192	-12.923	0.000
Thrombocytopenia	Finn RS 2015	Thrombocytopenia	0.169	0.103	0.265	-5.442	0.000
Thrombocytopenia			0.309	0.209	0.430	-3.009	0.003
Upper respiratory infection	Dickson MA 2013	Upper respiratory infection	0.034	0.005	0.208	-3.274	0.001
Upper respiratory infection	Cristofanilli M 2016	Upper respiratory infection	0.078	0.054	0.112	-12.303	0.000
Upper respiratory infection	Finn RS 2016	Upper respiratory infection	0.133	0.104	0.168	-13.416	0.000
Upper respiratory infection	Finn RS 2015	Upper respiratory infection	0.108	0.057	0.195	-5.968	0.000
Upper respiratory infection			0.101	0.069	0.146	-10.175	0.000

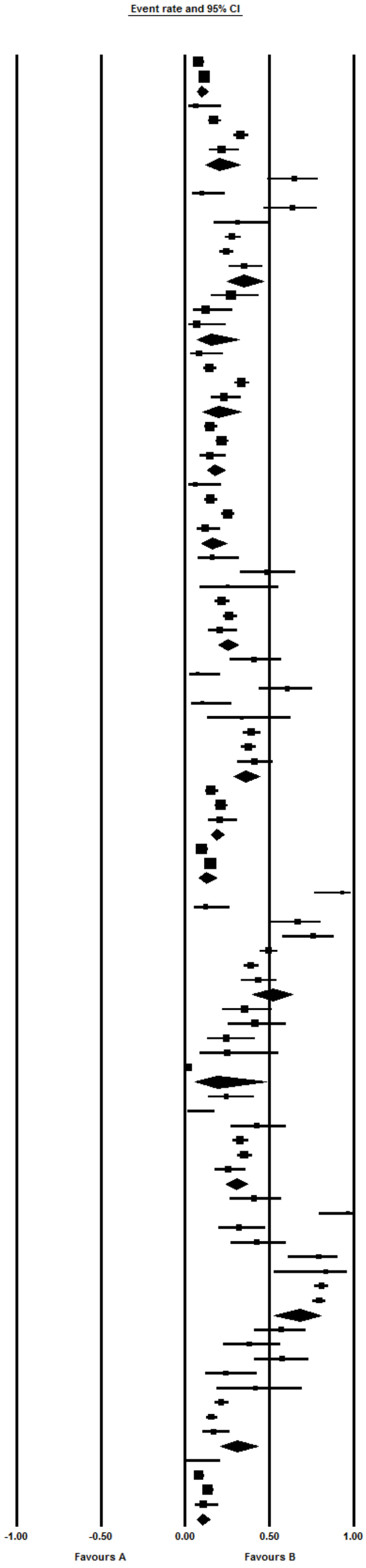

Supplementary Figure S2a. Grade≥3 adverse events in single arm groups (Fixed model).

Group by Subgroup within study	Study name	Adverse events	Event rate	Lower limit	Upper limit	Z-Value	p-Value
			Statistics for each study				
Alopecia	Cristofanilli M 2016	Alopecia	0.001	0.000	0.023	-4.620	0.000
Alopecia	Finn RS 2016	Alopecia	0.001	0.000	0.018	-4.799	0.000
Alopecia			0.001	0.000	0.009	-6.660	0.000
Anemia	DeMichele A 2014	Anemia	0.054	0.014	0.192	-3.937	0.000
Anemia	Dickson MA 2013	Anemia	0.172	0.074	0.353	-3.191	0.001
Anemia	Flaherty KT 2012	Anemia	0.098	0.037	0.233	-4.227	0.000
Anemia	Schwartz GK 2011	Anemia	0.030	0.004	0.186	-3.413	0.001
Anemia	Vaughn DJ 2015	Anemia	0.069	0.017	0.238	-3.552	0.000
Anemia	Cristofanilli M 2016	Anemia	0.029	0.016	0.053	-10.942	0.000
Anemia	Finn RS 2016	Anemia	0.054	0.036	0.079	-13.638	0.000
Anemia	Finn RS 2015	Anemia	0.060	0.025	0.137	-5.955	0.000
Anemia			0.056	0.043	0.073	-19.900	0.000
Arthralgia	Cristofanilli M 2016	Arthralgia	0.003	0.000	0.020	-5.832	0.000
Arthralgia	Finn RS 2016	Arthralgia	0.007	0.002	0.021	-8.614	0.000
Arthralgia	Finn RS 2015	Arthralgia	0.012	0.002	0.081	-4.380	0.000
Arthralgia			0.006	0.003	0.015	-11.241	0.000
Asthenia	Finn RS 2016	Asthenia	0.023	0.012	0.041	-11.788	0.000
Asthenia	Finn RS 2015	Asthenia	0.024	0.006	0.091	-5.171	0.000
Asthenia			0.023	0.013	0.040	-12.872	0.000
Back pain	Cristofanilli M 2016	Back pain	0.012	0.004	0.030	-8.839	0.000
Back pain	Finn RS 2016	Back pain	0.014	0.006	0.030	-10.438	0.000
Back pain	Finn RS 2015	Back pain	0.012	0.002	0.081	-4.380	0.000
Back pain			0.013	0.007	0.023	-14.360	0.000
Cough	Cristofanilli M 2016	Cough	0.001	0.000	0.023	-4.620	0.000
Cough	Finn RS 2016	Cough	0.001	0.000	0.018	-4.799	0.000
Cough			0.001	0.000	0.009	-6.660	0.000
Decreased appetite	Cristofanilli M 2016	Decreased appetite	0.009	0.003	0.027	-8.168	0.000
Decreased appetite	Finn RS 2016	Decreased appetite	0.007	0.002	0.021	-8.614	0.000
Decreased appetite	Finn RS 2015	Decreased appetite	0.012	0.002	0.081	-4.380	0.000
Decreased appetite			0.008	0.004	0.017	-12.642	0.000
Diarrhea	Tamura K 2016	Diarrhea	0.038	0.002	0.403	-2.232	0.026
Diarrhea	Cristofanilli M 2016	Diarrhea	0.001	0.000	0.023	-4.620	0.000
Diarrhea	Finn RS 2016	Diarrhea	0.014	0.006	0.030	-10.438	0.000
Diarrhea	Finn RS 2015	Diarrhea	0.036	0.012	0.106	-5.583	0.000
Diarrhea			0.017	0.009	0.031	-12.688	0.000
Dizziness	Cristofanilli M 2016	Dizziness	0.003	0.000	0.020	-5.832	0.000
Dizziness	Finn RS 2016	Dizziness	0.005	0.001	0.018	-7.617	0.000
Dizziness			0.004	0.001	0.012	-9.587	0.000
Fatigue	Dickson MA 2013	Fatigue	0.069	0.017	0.238	-3.552	0.000
Fatigue	Flaherty KT 2012	Fatigue	0.073	0.024	0.204	-4.234	0.000
Fatigue	Schwartz GK 2011	Fatigue	0.030	0.004	0.186	-3.413	0.001
Fatigue	Tamura K 2016	Fatigue	0.038	0.002	0.403	-2.232	0.026
Fatigue	Cristofanilli M 2016	Fatigue	0.023	0.012	0.046	-10.457	0.000
Fatigue	Finn RS 2016	Fatigue	0.018	0.009	0.036	-11.206	0.000
Fatigue	Finn RS 2015	Fatigue	0.048	0.018	0.121	-5.821	0.000
Fatigue			0.030	0.021	0.044	-17.563	0.000
Headache	Cristofanilli M 2016	Headache	0.006	0.001	0.023	-7.254	0.000
Headache	Finn RS 2016	Headache	0.002	0.000	0.016	-6.087	0.000
Headache			0.004	0.001	0.013	-9.438	0.000
Hot flushes	Cristofanilli M 2016	Hot flushes	0.001	0.000	0.023	-4.620	0.000
Hot flushes	Finn RS 2016	Hot flushes	0.001	0.000	0.018	-4.799	0.000
Hot flushes			0.001	0.000	0.009	-6.660	0.000
Insomnia	Cristofanilli M 2016	Insomnia	0.003	0.000	0.020	-5.832	0.000
Insomnia	Finn RS 2016	Insomnia	0.001	0.000	0.018	-4.799	0.000
Insomnia			0.002	0.000	0.010	-7.533	0.000
Mucositis	Cristofanilli M 2016	Mucositis	0.006	0.001	0.023	-7.254	0.000
Mucositis	Finn RS 2016	Mucositis	0.002	0.000	0.016	-6.087	0.000
Mucositis			0.004	0.001	0.013	-9.438	0.000
Musculoskeletal pain	Cristofanilli M 2016	Musculoskeletal pain	0.003	0.000	0.020	-5.832	0.000
Musculoskeletal pain	Finn RS 2015	Musculoskeletal pain	0.012	0.002	0.081	-4.380	0.000
Musculoskeletal pain			0.006	0.001	0.023	-7.224	0.000
Pain in extremity	Cristofanilli M 2016	Pain in extremity	0.001	0.000	0.023	-4.620	0.000
Pain in extremity	Finn RS 2016	Pain in extremity	0.002	0.000	0.016	-6.087	0.000
Pain in extremity			0.002	0.000	0.010	-7.637	0.000
Pyrexia	Cristofanilli M 2016	Pyrexia	0.003	0.000	0.020	-5.832	0.000
Pyrexia	Finn RS 2016	Pyrexia	0.001	0.000	0.018	-4.799	0.000
Pyrexia			0.002	0.000	0.010	-7.533	0.000
Rash	Cristofanilli M 2016	Rash	0.006	0.001	0.023	-7.254	0.000
Rash	Finn RS 2016	Rash	0.009	0.003	0.024	-9.359	0.000
Rash			0.008	0.003	0.017	-11.830	0.000
Vomiting	Schwartz GK 2011	Vomiting	0.030	0.004	0.186	-3.413	0.001
Vomiting	Cristofanilli M 2016	Vomiting	0.003	0.000	0.020	-5.832	0.000
Vomiting	Finn RS 2016	Vomiting	0.005	0.001	0.018	-7.617	0.000
Vomiting	Finn RS 2015	Vomiting	0.006	0.000	0.088	-3.608	0.000
Vomiting			0.006	0.003	0.016	-10.649	0.000

Event rate and 95% CI

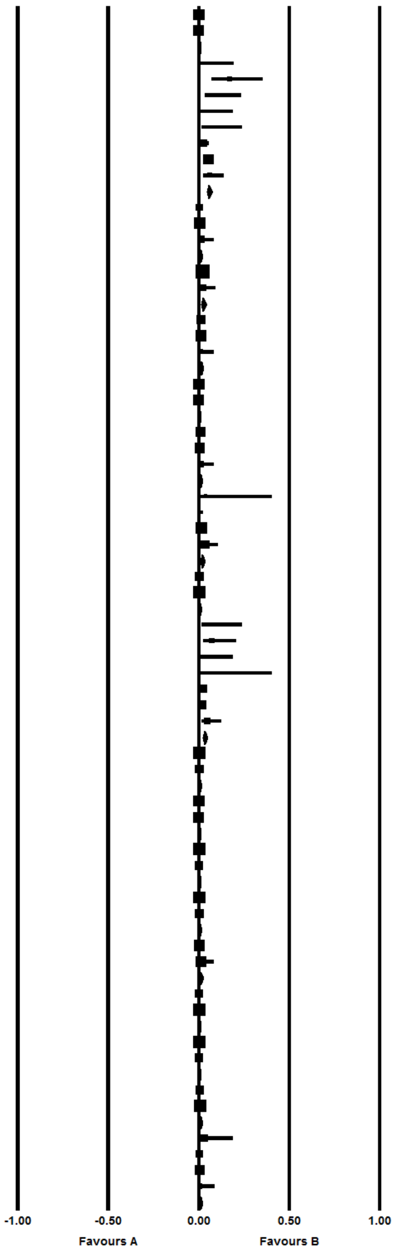

Supplementary Figure S2b. Grade≥3 adverse events in single arm groups (Random model).

Group by<br>Subgroup within study	Study name	Adverse events	Event rate	Lower limit	Upper limit	Z-Value	p-Value
			**Statistics for each study**				
Abdominal pain	Flaherty KT 2012	Abdominal pain	0.049	0.012	0.175	-4.097	0.000
Abdominal pain	Cristofanilli M 2016	Abdominal pain	0.006	0.001	0.023	-7.254	0.000
Abdominal pain	Finn RS 2016	Abdominal pain	0.009	0.003	0.024	-9.359	0.000
Abdominal pain			0.013	0.004	0.042	-7.136	0.000
Bone pain	Cristofanilli M 2016	Bone pain	0.006	0.001	0.023	-7.254	0.000
Bone pain	Finn RS 2015	Bone pain	0.024	0.006	0.091	-5.171	0.000
Bone pain			0.012	0.003	0.047	-6.134	0.000
Constipation	Dickson MA 2013	Constipation	0.034	0.005	0.208	-3.274	0.001
Constipation	Cristofanilli M 2016	Constipation	0.001	0.000	0.023	-4.620	0.000
Constipation	Finn RS 2016	Constipation	0.005	0.001	0.018	-7.617	0.000
Constipation			0.007	0.001	0.035	-5.903	0.000
Dyspnea	Flaherty KT 2012	Dyspnea	0.049	0.012	0.175	-4.097	0.000
Dyspnea	Cristofanilli M 2016	Dyspnea	0.003	0.000	0.020	-5.832	0.000
Dyspnea	Finn RS 2016	Dyspnea	0.011	0.005	0.027	-9.950	0.000
Dyspnea	Finn RS 2015	Dyspnea	0.024	0.006	0.091	-5.171	0.000
Dyspnea			0.016	0.006	0.041	-8.223	0.000
Leukopenia	DeMichele A 2014	Leukopenia	0.514	0.356	0.668	0.164	0.869
Leukopenia	Dickson MA 2013	Leukopenia	0.483	0.311	0.659	-0.186	0.853
Leukopenia	Flaherty KT 2012	Leukopenia	0.122	0.052	0.261	-4.136	0.000
Leukopenia	Schwartz GK 2011	Leukopenia	0.212	0.105	0.383	-3.082	0.002
Leukopenia	Vaughn DJ 2015	Leukopenia	0.379	0.224	0.564	-1.287	0.198
Leukopenia	Tamura K 2016	Leukopenia	0.417	0.185	0.692	-0.575	0.566
Leukopenia	Cristofanilli M 2016	Leukopenia	0.275	0.231	0.325	-8.028	0.000
Leukopenia	Finn RS 2016	Leukopenia	0.248	0.210	0.290	-10.103	0.000
Leukopenia	Finn RS 2015	Leukopenia	0.193	0.122	0.292	-5.147	0.000
Leukopenia			0.294	0.232	0.366	-5.292	0.000
Nausea	Flaherty KT 2012	Nausea	0.049	0.012	0.175	-4.097	0.000
Nausea	Schwartz GK 2011	Nausea	0.030	0.004	0.186	-3.413	0.001
Nausea	Cristofanilli M 2016	Nausea	0.001	0.000	0.023	-4.620	0.000
Nausea	Finn RS 2016	Nausea	0.002	0.000	0.016	-6.087	0.000
Nausea	Finn RS 2015	Nausea	0.024	0.006	0.091	-5.171	0.000
Nausea			0.013	0.004	0.046	-6.557	0.000
Neutropenia	DeMichele A 2014	Neutropenia	0.541	0.381	0.692	0.493	0.622
Neutropenia	Dickson MA 2013	Neutropenia	0.517	0.341	0.689	0.186	0.853
Neutropenia	Flaherty KT 2012	Neutropenia	0.317	0.194	0.473	-2.286	0.022
Neutropenia	Schwartz GK 2011	Neutropenia	0.242	0.126	0.415	-2.805	0.005
Neutropenia	Vaughn DJ 2015	Neutropenia	0.448	0.281	0.628	-0.556	0.578
Neutropenia	Tamura K 2016	Neutropenia	0.583	0.308	0.815	0.575	0.566
Neutropenia	Cristofanilli M 2016	Neutropenia	0.646	0.594	0.695	5.356	0.000
Neutropenia	Finn RS 2016	Neutropenia	0.664	0.619	0.707	6.796	0.000
Neutropenia	Finn RS 2015	Neutropenia	0.542	0.435	0.646	0.767	0.443
Neutropenia			0.516	0.427	0.603	0.347	0.729
Thrombocytopenia	DeMichele A 2014	Thrombocytopenia	0.189	0.093	0.347	-3.467	0.001
Thrombocytopenia	Dickson MA 2013	Thrombocytopenia	0.310	0.170	0.497	-1.989	0.047
Thrombocytopenia	Schwartz GK 2011	Thrombocytopenia	0.091	0.030	0.247	-3.803	0.000
Thrombocytopenia	Vaughn DJ 2015	Thrombocytopenia	0.172	0.074	0.353	-3.191	0.001
Thrombocytopenia	Tamura K 2016	Thrombocytopenia	0.083	0.012	0.413	-2.296	0.022
Thrombocytopenia	Cristofanilli M 2016	Thrombocytopenia	0.023	0.012	0.046	-10.457	0.000
Thrombocytopenia	Finn RS 2016	Thrombocytopenia	0.016	0.008	0.033	-10.851	0.000
Thrombocytopenia	Finn RS 2015	Thrombocytopenia	0.024	0.006	0.091	-5.171	0.000
Thrombocytopenia			0.075	0.029	0.181	-4.914	0.000
Upper respiratory infection	Dickson MA 2013	Upper respiratory infection	0.034	0.005	0.208	-3.274	0.001
Upper respiratory infection	Cristofanilli M 2016	Upper respiratory infection	0.006	0.001	0.023	-7.254	0.000
Upper respiratory infection	Finn RS 2016	Upper respiratory infection	0.001	0.000	0.018	-4.799	0.000
Upper respiratory infection			0.007	0.001	0.037	-5.772	0.000

Event rate and 95% CI

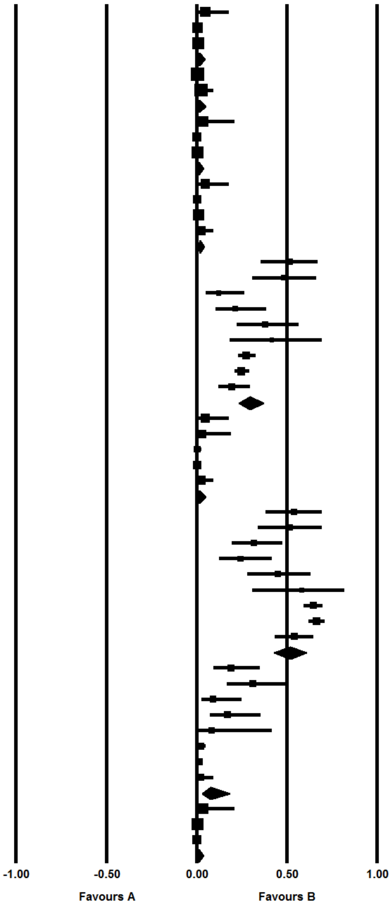

Supplementary Figure S3a. All grade adverse events in controlled groups (Fixed model).

Group by / Subgroup within study	Study name	Adverse events	Statistics for each study: Odds ratio	Lower limit	Upper limit	Z-Value	p-Value
Abdominal pain	Cristofanilli M 2016	Abdominal pain	1.375	0.650	2.911	0.833	0.405
Abdominal pain	Finn RS 2016	Abdominal pain	2.221	1.157	4.262	2.399	0.016
Abdominal pain			1.807	1.105	2.955	2.357	0.018
Alopecia	Cristofanilli M 2016	Alopecia	2.958	1.509	5.797	3.159	0.002
Alopecia	Finn RS 2016	Alopecia	2.618	1.734	3.951	4.581	0.000
Alopecia	Finn RS 2015	Alopecia	10.385	2.321	46.455	3.062	0.002
Alopecia			2.903	2.062	4.086	6.108	0.000
Anemia	Cristofanilli M 2016	Anemia	3.105	1.824	5.284	4.176	0.000
Anemia	Finn RS 2016	Anemia	3.207	1.929	5.331	4.493	0.000
Anemia	Finn RS 2015	Anemia	7.733	2.809	21.287	3.960	0.000
Anemia			3.504	2.481	4.950	7.116	0.000
Arthralgia	Cristofanilli M 2016	Arthralgia	0.889	0.534	1.481	-0.452	0.651
Arthralgia	Finn RS 2016	Arthralgia	0.980	0.697	1.378	-0.116	0.908
Arthralgia	Finn RS 2015	Arthralgia	1.608	0.722	3.582	1.163	0.245
Arthralgia			1.008	0.772	1.317	0.060	0.952
Asthenia	Finn RS 2016	Asthenia	1.532	0.950	2.472	1.748	0.081
Asthenia	Finn RS 2015	Asthenia	3.769	1.010	14.067	1.974	0.048
Asthenia			1.702	1.085	2.668	2.317	0.021
Back pain	Cristofanilli M 2016	Back pain	0.855	0.520	1.407	-0.615	0.538
Back pain	Finn RS 2016	Back pain	1.000	0.676	1.479	0.000	1.000
Back pain	Finn RS 2015	Back pain	0.915	0.384	2.181	-0.199	0.842
Back pain			0.939	0.703	1.255	-0.425	0.671
Constipation	Cristofanilli M 2016	Constipation	1.270	0.778	2.075	0.956	0.339
Constipation	Finn RS 2016	Constipation	1.328	0.860	2.051	1.281	0.200
Constipation	Finn RS 2015	Constipation	1.370	0.494	3.799	0.605	0.545
Constipation			1.309	0.960	1.784	1.701	0.089
Cough	Cristofanilli M 2016	Cough	1.183	0.691	2.024	0.612	0.540
Cough	Finn RS 2016	Cough	1.429	0.959	2.128	1.753	0.080
Cough	Finn RS 2015	Cough	1.182	0.441	3.168	0.331	0.740
Cough			1.320	0.974	1.790	1.789	0.074
Decreased appetite	Cristofanilli M 2016	Decreased appetite	2.003	1.076	3.727	2.192	0.028
Decreased appetite	Finn RS 2016	Decreased appetite	1.763	1.039	2.992	2.103	0.035
Decreased appetite	Finn RS 2015	Decreased appetite	2.674	0.906	7.895	1.781	0.075
Decreased appetite			1.944	1.333	2.835	3.454	0.001
Diarrhea	Cristofanilli M 2016	Diarrhea	1.195	0.753	1.896	0.754	0.451
Diarrhea	Finn RS 2016	Diarrhea	1.472	0.992	2.184	1.922	0.055
Diarrhea	Finn RS 2015	Diarrhea	2.222	0.898	5.495	1.728	0.084
Diarrhea			1.416	1.065	1.883	2.396	0.017
Dizziness	Cristofanilli M 2016	Dizziness	1.315	0.715	2.418	0.881	0.378
Dizziness	Finn RS 2016	Dizziness	0.947	0.600	1.494	-0.234	0.815
Dizziness	Finn RS 2015	Dizziness	2.631	0.672	10.305	1.389	0.165
Dizziness			1.132	0.795	1.610	0.688	0.492
Dyspnea	Cristofanilli M 2016	Dyspnea	1.480	0.782	2.802	1.204	0.229
Dyspnea	Finn RS 2016	Dyspnea	1.117	0.702	1.779	0.468	0.640
Dyspnea	Finn RS 2015	Dyspnea	2.198	0.791	6.107	1.510	0.131
Dyspnea			1.320	0.927	1.878	1.542	0.123
Fatigue	Cristofanilli M 2016	Fatigue	1.614	1.087	2.396	2.372	0.018
Fatigue	Finn RS 2016	Fatigue	1.576	1.109	2.241	2.534	0.011
Fatigue	Finn RS 2015	Fatigue	2.274	1.146	4.514	2.349	0.019
Fatigue			1.667	1.304	2.131	4.081	0.000
Headache	Cristofanilli M 2016	Headache	1.272	0.807	2.003	1.036	0.300
Headache	Finn RS 2016	Headache	0.770	0.529	1.121	-1.366	0.172
Headache	Finn RS 2015	Headache	1.458	0.562	3.784	0.774	0.439
Headache			0.979	0.742	1.291	-0.151	0.880
Insomnia	Cristofanilli M 2016	Insomnia	1.410	0.709	2.805	0.980	0.327
Insomnia	Finn RS 2016	Insomnia	1.316	0.810	2.139	1.109	0.267
Insomnia			1.347	0.906	2.002	1.471	0.141
Leukopenia	Cristofanilli M 2016	Leukopenia	23.165	10.563	50.799	7.844	0.000
Leukopenia	Finn RS 2016	Leukopenia	27.706	11.187	68.613	7.179	0.000
Leukopenia	Finn RS 2015	Leukopenia	28.723	6.605	124.903	4.477	0.000
Leukopenia			25.502	14.707	44.220	11.533	0.000
Mucositis	Cristofanilli M 2016	Mucositis	5.980	2.110	16.949	3.365	0.001
Mucositis	Finn RS 2016	Mucositis	2.908	1.569	5.388	3.391	0.001
Mucositis	Finn RS 2015	Mucositis	5.137	1.088	24.251	2.067	0.039
Mucositis			3.649	2.208	6.030	5.052	0.000
Myalgia	Finn RS 2016	Myalgia	1.369	0.796	2.353	1.137	0.256
Myalgia	Finn RS 2015	Myalgia	1.751	0.560	5.478	0.963	0.335
Myalgia			1.433	0.878	2.337	1.440	0.150
Nausea	Cristofanilli M 2016	Nausea	1.278	0.853	1.915	1.192	0.233
Nausea	Finn RS 2016	Nausea	1.532	1.072	2.189	2.339	0.019
Nausea	Finn RS 2015	Nausea	2.269	0.991	5.196	1.939	0.053
Nausea			1.479	1.147	1.909	3.014	0.003
Neutropenia	Cristofanilli M 2016	Neutropenia	116.955	49.613	275.700	10.884	0.000
Neutropenia	Finn RS 2016	Neutropenia	57.633	32.006	103.779	13.509	0.000
Neutropenia			72.277	44.499	117.396	17.297	0.000
Pain in extremity	Cristofanilli M 2016	Pain in extremity	1.024	0.586	1.787	0.083	0.934
Pain in extremity	Finn RS 2016	Pain in extremity	0.849	0.551	1.306	-0.746	0.456
Pain in extremity	Finn RS 2015	Pain in extremity	1.262	0.417	3.819	0.412	0.680
Pain in extremity			0.937	0.676	1.298	-0.394	0.694
Pyrexia	Cristofanilli M 2016	Pyrexia	2.242	1.058	4.751	2.107	0.035
Pyrexia	Finn RS 2016	Pyrexia	1.511	0.873	2.614	1.474	0.140
Pyrexia			1.733	1.113	2.699	2.433	0.015
Thrombocytopenia	Cristofanilli M 2016	Thrombocytopenia	93.055	5.728	1511.626	3.187	0.001
Thrombocytopenia	Finn RS 2016	Thrombocytopenia	13.432	4.178	43.186	4.359	0.000
Thrombocytopenia	Finn RS 2015	Thrombocytopenia	15.420	1.976	120.356	2.609	0.009
Thrombocytopenia			17.359	6.686	45.065	5.863	0.000
Upper respiratory infection	Cristofanilli M 2016		1.132	0.559	2.294	0.344	0.731
Upper respiratory infection	Finn RS 2016		1.208	0.734	1.987	0.742	0.458
Upper respiratory infection	Finn RS 2015		1.561	0.953	21.823	1.900	0.057
Upper respiratory infection			1.287	0.868	1.909	1.257	0.209

Odds ratio and 95% CI

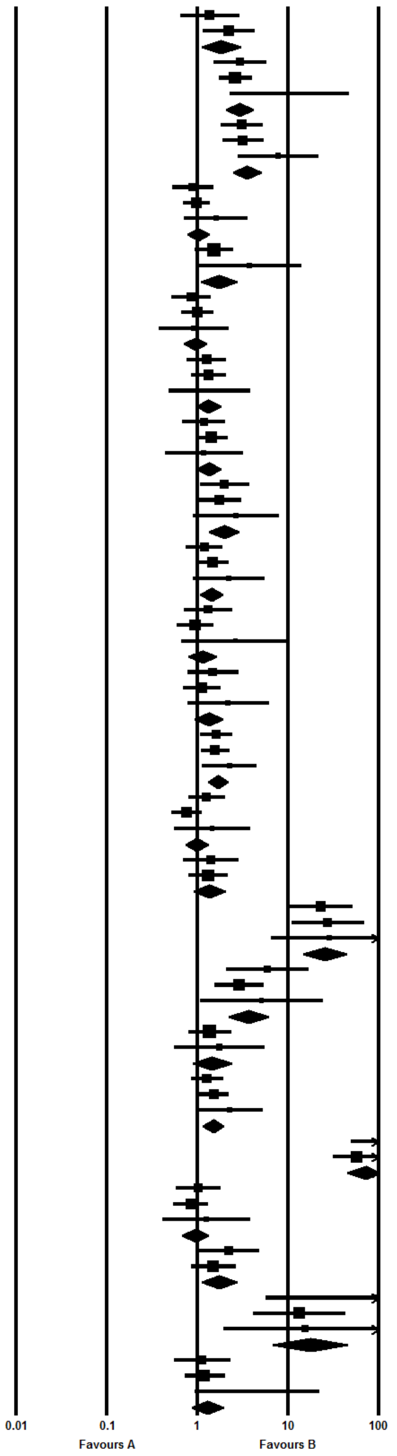

Supplementary Figure S3b. All grade adverse events in controlled groups (Random model).

Group by / Subgroup within study	Study name	Adverse events	Odds ratio	Lower limit	Upper limit	Z-Value	p-Value
Hot flushes	Cristofanilli M 2016	Hot flushes	0.895	0.546	1.468	-0.439	0.661
Hot flushes	Finn RS 2016	Hot flushes	0.600	0.416	0.865	-2.738	0.006
Hot flushes	Finn RS 2015	Hot flushes	1.946	0.810	4.674	1.490	0.136
Hot flushes			0.904	0.516	1.584	-0.353	0.724
Rash	Cristofanilli M 2016	Rash	3.214	1.544	6.690	3.122	0.002
Rash	Finn RS 2016	Rash	1.632	1.014	2.626	2.016	0.044
Rash			2.157	1.122	4.149	2.304	0.021
Vomiting	Cristofanilli M 2016	Vomiting	1.188	0.714	1.978	0.664	0.507
Vomiting	Finn RS 2016	Vomiting	0.920	0.595	1.423	-0.374	0.708
Vomiting	Finn RS 2015	Vomiting	4.169	1.129	15.395	2.142	0.032
Vomiting			1.267	0.722	2.224	0.824	0.410

Statistics for each study

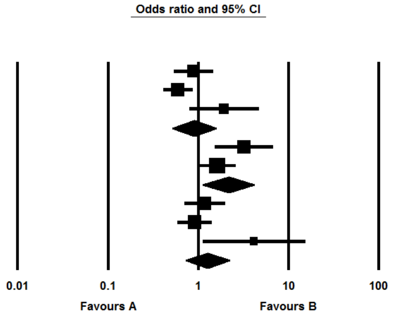

Supplementary Figure S4a. Grade≥3 adverse events in controlled groups (Fixed model).

Group by / Subgroup within study	Study name	Adverse events	Odds ratio	Lower limit	Upper limit	Z-Value	p-Value
Abdominal pain	Cristofanilli M 2016	Abdominal pain	0.997	0.090	11.073	-0.002	0.998
Abdominal pain	Finn RS 2016	Abdominal pain	4.546	0.244	84.811	1.014	0.310
Abdominal pain			1.839	0.287	11.806	0.643	0.521
Anemia	Cristofanilli M 2016	Anemia	1.682	0.457	6.191	0.782	0.434
Anemia	Finn RS 2016	Anemia	3.114	1.067	9.089	2.079	0.038
Anemia	Finn RS 2015	Anemia	4.872	0.556	42.675	1.430	0.153
Anemia			2.654	1.225	5.750	2.474	0.013
Arthralgia	Cristofanilli M 2016	Arthralgia	1.502	0.061	37.067	0.249	0.804
Arthralgia	Finn RS 2016	Arthralgia	1.503	0.155	14.536	0.352	0.725
Arthralgia	Finn RS 2015	Arthralgia	0.457	0.041	5.147	-0.633	0.526
Arthralgia			0.969	0.223	4.217	-0.042	0.966
Asthenia	Finn RS 2016	Asthenia	10.754	0.627	184.359	1.638	0.101
Asthenia	Finn RS 2015	Asthenia	4.755	0.225	100.621	1.001	0.317
Asthenia			7.362	0.920	58.917	1.881	0.060
Bone pain	Cristofanilli M 2016	Bone pain	0.496	0.069	3.549	-0.699	0.485
Bone pain	Finn RS 2015	Bone pain	4.755	0.225	100.621	1.001	0.317
Bone pain			0.963	0.184	5.036	-0.045	0.964
Decreased appetite	Cristofanilli M 2016	Decreased appetite	1.500	0.155	14.528	0.350	0.726
Decreased appetite	Finn RS 2016	Decreased appetite	0.528	0.181	68.595	0.833	0.405
Decreased appetite	Finn RS 2015	Decreased appetite	2.818	0.113	70.223	0.632	0.528
Decreased appetite			2.218	0.460	10.691	0.993	0.321
Diarrhea	Cristofanilli M 2016	Diarrhea	0.165	0.007	4.083	-1.100	0.271
Diarrhea	Finn RS 2016	Diarrhea	1.000	0.248	4.036	0.000	1.000
Diarrhea	Finn RS 2015	Diarrhea	6.739	0.342	132.620	1.255	0.209
Diarrhea			1.057	0.326	3.424	0.092	0.927
Dizziness	Cristofanilli M 2016	Dizziness	1.502	0.061	37.067	0.249	0.804
Dizziness	Finn RS 2016	Dizziness	2.514	0.120	52.592	0.594	0.552
Dizziness			1.970	0.217	17.889	0.602	0.547
Dyspnea	Cristofanilli M 2016	Dyspnea	0.247	0.022	2.744	-1.138	0.255
Dyspnea	Finn RS 2016	Dyspnea	0.831	0.197	3.511	-0.251	0.802
Dyspnea	Finn RS 2015	Dyspnea	1.877	0.167	21.120	0.510	0.610
Dyspnea			0.763	0.254	2.295	-0.481	0.631
Fatigue	Cristofanilli M 2016	Fatigue	2.018	0.424	9.606	0.882	0.378
Fatigue	Finn RS 2016	Fatigue	4.055	0.504	32.626	1.316	0.188
Fatigue	Finn RS 2015	Fatigue	3.848	0.421	35.212	1.193	0.233
Fatigue			2.852	0.961	8.466	1.888	0.059
Leukopenia	Cristofanilli M 2016	Leukopenia	32.300	7.855	132.817	4.817	0.000
Leukopenia	Finn RS 2016	Leukopenia	147.003	9.090	2377.237	3.514	0.000
Leukopenia	Finn RS 2015	Leukopenia	37.889	2.231	643.556	2.515	0.012
Leukopenia			42.988	13.589	135.989	6.401	0.000
Mucositis	Cristofanilli M 2016	Mucositis	2.511	0.120	52.589	0.593	0.553
Mucositis	Finn RS 2016	Mucositis	1.505	0.061	37.096	0.250	0.803
Mucositis			1.970	0.217	17.891	0.602	0.547
Musculoskeletal pain	Cristofanilli M 2016	Musculoskeletal pain	0.497	0.031	7.996	-0.493	0.622
Musculoskeletal pain	Finn RS 2015	Musculoskeletal pain	2.818	0.113	70.223	0.632	0.528
Musculoskeletal pain			1.043	0.128	8.539	0.040	0.968
Nausea	Cristofanilli M 2016	Nausea	0.165	0.007	4.083	-1.100	0.271
Nausea	Finn RS 2016	Nausea	0.123	0.014	1.107	-1.869	0.062
Nausea	Finn RS 2015	Nausea	1.877	0.167	21.120	0.510	0.610
Nausea			0.348	0.082	1.484	-1.426	0.154
Neutropenia	Cristofanilli M 2016	Neutropenia	312.566	43.240	2259.396	5.692	0.000
Neutropenia	Finn RS 2016	Neutropenia	144.530	45.480	459.299	8.431	0.000
Neutropenia	Finn RS 2015	Neutropenia	90.000	11.945	678.124	4.367	0.000
Neutropenia			154.215	63.023	377.360	11.035	0.000
Pain in extremity	Cristofanilli M 2016	Pain in extremity	0.070	0.004	1.365	-1.755	0.079
Pain in extremity	Finn RS 2016	Pain in extremity	0.165	0.017	1.593	-1.558	0.119
Pain in extremity			0.120	0.020	0.729	-2.303	0.021
Rash	Cristofanilli M 2016	Rash	2.511	0.120	52.589	0.593	0.553
Rash	Finn RS 2016	Rash	2.009	0.223	18.082	0.622	0.534
Rash			2.169	0.365	12.875	0.852	0.394
Thrombocytopenia	Cristofanilli M 2016	Thrombocytopenia	8.689	0.499	151.427	1.483	0.138
Thrombocytopenia	Finn RS 2016	Thrombocytopenia	7.629	0.434	134.177	1.389	0.165
Thrombocytopenia	Finn RS 2015	Thrombocytopenia	4.755	0.225	100.621	1.001	0.317
Thrombocytopenia			6.909	1.279	37.329	2.246	0.025
Vomiting	Cristofanilli M 2016	Vomiting	0.497	0.031	7.996	-0.493	0.622
Vomiting	Finn RS 2016	Vomiting	0.330	0.055	1.991	-1.208	0.227
Vomiting	Finn RS 2015	Vomiting	0.305	0.012	7.610	-0.723	0.470
Vomiting			0.359	0.092	1.409	-1.468	0.142

Odds ratio and 95% CI

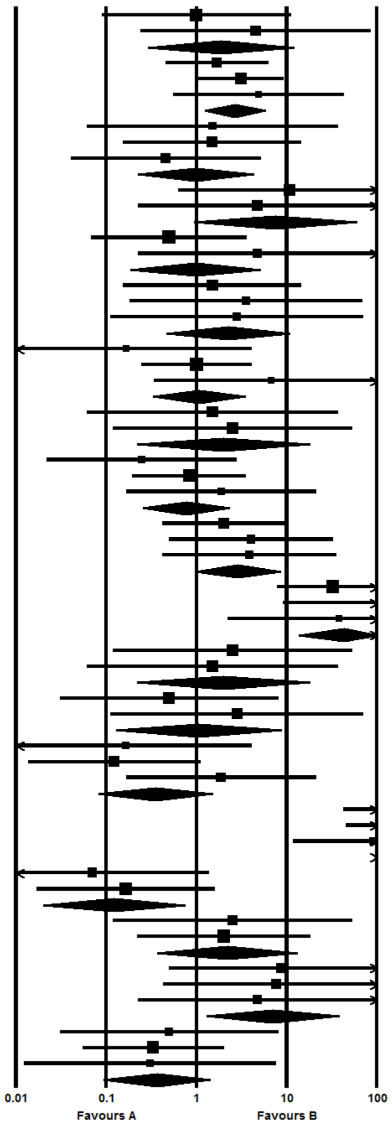

Supplementary Figure S4b. Grade≥3 adverse events in controlled groups (Random model).

Group by / Subgroup within study	Study name	Adverse events	Odds ratio	Lower limit	Upper limit	Z-Value	p-Value
Headache	Cristofanilli M 2016	Headache	2.511	0.120	52.589	0.593	0.553
Headache	Finn RS 2016	Headache	0.123	0.014	1.107	-1.869	0.062
Headache			0.459	0.024	8.615	-0.520	0.603

Odds ratio and 95% CI

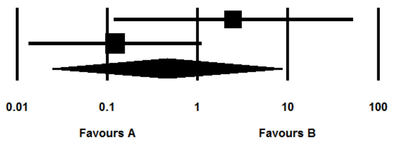

Supplement: Supplementary file 1 [file CAM4-8-1389-s001.pdf]
